# Physicochemical Characterization, Biocompatibility, and Antibacterial Properties of CMC/PVA/*Calendula officinalis* Films for Biomedical Applications

**DOI:** 10.3390/polym15061454

**Published:** 2023-03-14

**Authors:** Wen-Hsin Huang, Chia-Yi Hung, Pao-Chang Chiang, Hsiang Lee, I-Ting Lin, Pin-Chuang Lai, Ya-Hui Chan, Sheng-Wei Feng

**Affiliations:** 1 Department of Stomatology, MacKay Memorial Hospital, Taipei 104, Taiwan; 2 School of Dentistry and Graduate Institute of Dental Science, National Defense Medical Center, Taipei 114, Taiwan; 3 School of Dentistry, College of Oral Medicine, Taipei Medical University, Taipei 110, Taiwan; 4 Pro Hallmarks Taiwan Ltd., Taipei 114, Taiwan; 5 Department of Periodontics, University of Missouri-Kansas City School of Dentistry, Kansas City, MO 64108, USA; 6 School of Oral Hygiene, College of Oral Medicine, Taipei Medical University, Taipei 110, Taiwan; 7 Division of Prosthodontics, Department of Dentistry, Taipei Medical University Hospital, Taipei 110, Taiwan

**Keywords:** carboxymethyl cellulose, polyvinyl alcohol, antibacterial property

## Abstract

This study reports a carboxymethyl cellulose (CMC)/polyvinyl alcohol (PVA) composite film that incorporates *Calendula officinalis* (CO) extract for biomedical applications. The morphological, physical, mechanical, hydrophilic, biological, and antibacterial properties of CMC/PVA composite films with various CO concentrations (0.1%, 1%, 2.5%, 4%, and 5%) are fully investigated using different experiments. The surface morphology and structure of the composite films are significantly affected by higher CO concentrations. X-ray diffraction (XRD) and Fourier transform infrared spectrometry (FTIR) analyses confirm the structural interactions among CMC, PVA, and CO. After CO is incorporated, the tensile strength and elongation upon the breaking of the films decrease significantly. The addition of CO significantly reduces the ultimate tensile strength of the composite films from 42.8 to 13.2 MPa. Furthermore, by increasing the concentration of CO to 0.75%, the contact angle is decreased from 15.8° to 10.9°. The MTT [3-(4,5-dimethylthiazol-2-yl)-2,5-diphenyl tetrazolium bromide] assay reveals that the CMC/PVA/CO-2.5% and CMC/PVA/CO-4% composite films are non-cytotoxic to human skin fibroblast cells, which is favorable for cell proliferation. Remarkably, 2.5% and 4% CO incorporation significantly improve the inhibition ability of the CMC/PVA composite films against Staphylococcus aureus and Escherichia coli. In summary, CMC/PVA composite films containing 2.5% CO exhibit the functional properties for wound healing and biomedical engineering applications.

## 1. Introduction

Wounds in skin tissues are usually caused by thermal, chemical, and traumatic injuries. Wound healing is one of the most complex and dynamic processes in the human body, involving homeostasis, inflammation, proliferation, and the remodeling of skin tissue [1]. However, wound healing is easily affected and delayed in patients with diabetes mellitus and complicated systematic health conditions [2,3]. In addition, delays to wound healing significantly increase the risk of wound infection, which results in immune responses, tissue inflammation, and damage. Thus, the development of wound dressings with biocompatible and antimicrobial properties is important to accelerate the wound healing process [4]. Recently, biopolymer-based wound dressings prepared by combining natural and synthetic polymers have attracted considerable attention.

Carboxymethyl cellulose (CMC) is a natural polymer and water-soluble derivative of cellulose. CMC has been widely used in the pharmaceutical, cosmetic, and food industries because of its excellent film-forming ability, high swelling ability, biocompatibility, biodegradability, hydrophilicity, cost-effectiveness, and stable internal network structure properties [5,6]. The hydrophilic groups (CH_2_COO−) of CMC enhance hydrogen bonding and bind to the hydroxyl groups of the glucopyranose chain of cellulose that constitutes its backbone [6,7]. Furthermore, CMC is a pH- and ionic strength-sensitive polysaccharide. As a polyanionic polymer, CMC has been found to possess bioadhesive properties and can strongly attach to the mucosal surfaces of the oral cavity and gastrointestinal tract [7]. The mucoadhesive properties of CMC allow for prolonged contact time in the specific tissues, thus enhancing bioavailability and preventing degradation for drug delivery applications [8]. In addition, CMC has also been shown to adhere to the mucous membrane and skin easily, which is beneficial for wound healing and skin regeneration applications [9]. These properties are crucial for the preparation of CMC scaffolds, hydrogels, and films for biomedical applications. However, the weaknesses of CMC include its poor mechanical strength, stability, and barrier properties [10,11]. To achieve significant effects, blending with other water-soluble polymers is a promising solution to overcome the disadvantages [12].

Among the most common synthetic polymers, poly(vinyl alcohol) (PVA) is a water-soluble semi-crystalline polymer that is predominantly composed of carbon chains. PVA is used in numerous biomedical and pharmaceutical applications because it possesses many useful characteristics, including biodegradability, flexibility, hydrophilicity, cell-adhesive properties, controlled tensile strength, non-toxicity, and an excellent film-forming ability [12,13]. PVA is prepared through the polymerization of vinyl acetate followed by partial hydrolysis. PVA is a non-ionic polymer with an odorless and translucent nature, and it has the ability to form an oxygen barrier [14]. Both CMC and PVA are biodegradable and biocompatible. After blending CMC with PVA, the strong hydrogen bonding between the hydroxyl groups results in a remarkable improvement in the mechanical properties [12,15,16]. In addition, recent studies have suggested that the incorporation of bio-compounds can improve the bioactivity and antimicrobial activity of composite films [12,16].

Essential oils and extracts of herbal materials are known to be powerful antioxidant and antimicrobial agents in biomedical applications [17,18,19]. The surface modification of metallic materials with peppermint essential oils was demonstrated to have bacteriostatic effects on Gram-positive bacteria and prevent biofilm formation [20]. *Calendula officinalis* (CO) is a medicinal herb also known as pot marigold [21,22]. CO flower extract consists of carotenes, flavonoids, terpenoids, triterpenoids, polyphenols, phenolic acids, quinines, coumarins, and other constituents [23,24]. It has been demonstrated to possess anti-inflammatory, antioxidant, antimicrobial, antifungal, free radical inhibitory, and wound-healing activities [25,26,27,28]. In comparison to cinnamon and clove essential oils, CO flower extracts have additional biological effects, including the enhancement of cell proliferation/migration and the promotion of collagen metabolism and angiogenesis at the wound site [27,28]. Furthermore, CO flower extracts have been applied for the treatment of skin burns, ulcerations, inflammation, and wounds [22,26]. The pharmacological activity of CO extract can be enhanced via incorporation into a polymeric matrix with controlled release over time for wound healing [21,27].

However, CMC/PVA/CO composite films have not yet been investigated. In addition, the optimal contents and proportions of CMC/PVA/CO composite films to achieve better mechanical, bioactive, and antimicrobial properties remain unclear. Thus, the aims of the present study are to develop novel CMC/PVA/CO composite films using the solution casting method and investigate the morphological, physical, mechanical, biological, and antimicrobial properties of the composite films.

## 2. Materials and Methods

### 2.1. Materials

Sodium CMC (high viscosity; MW: 700 kDa) and PVA (MW: 9000 Da) were purchased from Sigma-Aldrich (St. Louis, MO, USA). All other chemicals and solvents used in the present study were of analytical grade and did not require further purification. Bacterial strains of Staphylococcus aureus (ATCC 25923) and Escherichia coli (ATCC 25922) were acquired from the American Type Culture Collection.

### 2.2. Preparation of CMC/PVA/CO Films

CMC (2%; *w*/*v*) and PVA (5%; *w*/*v*) were dissolved in distilled water via continuous magnetic stirring for 60 min at 80 °C. The obtained CMC and PVA solutions were mixed (1:1; *v*/*v*) under mechanical stirring for 30 min. Different ratios (0%, 0.1%, 1%, 2.5%, 4%, and 5%) of CO extract (Cheng Yi Chemical Co, Taiwan) containing glycerol and TWEEN-80 relative to the CMC/PVA mixture were prepared. The composition ratio of CO extract, glycerol, and TWEEN-80 for the 5% group was 95:1:4 (*v*/*v*), respectively. By adjusting the composition ratios of CO extract, the other experimental groups with different ratios (4%, 2.5%, 1%, 0.1%, and 0%) of CO extract containing glycerol and TWEEN-80 relative to the CMC/PVA mixture could be obtained. The CMC/PVA/CO films were developed using the solution casting method [17]. Briefly, the CMC/PVA/CO mixed solutions were magnetically stirred for 30 min and placed under a vacuum for 10 min. After obtaining a homogeneous solution, the final solutions were poured into a Petri dish with a diameter of 10 cm and dried in a vacuum oven at 40 °C for 24 h to obtain the CMC/PVA/CO composite films.

### 2.3. Scanning Electron Microscopy

The surface morphology of the CMC/PVA/CO composite films was examined using scanning electron microscopy (SEM; Hitachi SU-3500, Hitachi High Technologies, Minato-ku, Tokyo, Japan) at an accelerating voltage of 5–20 kV and a working distance of 5 mm [29]. Prior to observation, all of the CMC/PVA/CO composite films were coated with a thin layer of gold for 10 min under a high vacuum at 10 kV for 90 s. Moreover, the cross-sectional viewpoints of the composite films were captured at 90°, relative to the electron beam.

### 2.4. X-ray Diffraction (XRD) and Fourier Transform Infrared Spectrometry (FTIR)

The crystalline nature of the CMC/PVA/CO films was characterized using X-ray diffraction (XRD; Empyrean, PANalytical BV, Almelo, The Netherlands) with Cu Kα radiation (λ = 0.154 nm) in the range of 2*θ* = 5°–90° and a step size and scan speed of 0.05° and 2°/min, respectively.

The chemical compositions of the CMC/PVA/CO films were characterized using Fourier transform infrared (FTIR) spectroscopy (Spotlight 200i Sp2 with an AutoATR System, PerkinElmer, Waltham, MA, USA). Spectra in the range of 4000–400 cm^–1^ were recorded in transmission mode with a resolution of 0.5 cm^–1^.

### 2.5. Thickness and Mechanical Properties

The thickness of the CMC/PVA/CO composite films was measured using a hand-held electronic digital micrometer (Mitutoyo, Japan) with an accuracy of 0.01 mm. Composite films with smooth and uniform thickness were selected for measurement. The mean values were calculated from five random positions on each film.

The mechanical properties of the CMC/PVA/CO composite films, including the ultimate tensile strength (TS) and elongation rate at break (EB), were determined using a universal testing machine (AGX-V, SHIMADZU, Kyoto, Japan). Prior to testing, the composite films were cut into rectangular strips (5 × 50 mm^2^) and mounted in the machine. The initial grip separation and cross-head speed were set at 25 mm and 1 mm/min, respectively. Finally, the TS (MPa) and EB (%) were measured for each composite film according to previous studies [17,30].

### 2.6. Contact Angle

The differences in the surface wettability of the CMC/PVA/CO composite films were measured using optical measurements of the static contact angle of water. Briefly, a 5 μL water drop was slowly added to the film surface and recorded using a video contact angle system (FTA-32, First Ten Angstroms, Portsmouth, VA, USA). After drawing the height and width of the drops on the film surface, the contact angle value was automatically calculated in the system [29].

### 2.7. In Vitro Cell Culture and Cell Viability

A human skin fibroblast cell line (CCD-966SK) was used for the cytocompatibility test. CCD-966SK cells were cultured and maintained in Dulbecco’s Modified Eagle Medium (DMEM) supplemented with 10% fetal bovine serum (FBS), 2 mM L-glutamine, 100 U/mL penicillin, 100 mg/mL streptomycin mixed antibiotics, and 1 mM sodium pyruvate under standard growth conditions in a 5% CO_2_ incubator.

The MTT (3-(4,5-dimethylthiazol-2-yl)-2,5-diphenyltetrazolium bromide) (Sigma-Aldrich, St. Louis, MO, USA) assay was performed to determine the CCD-966SK cell viability [31]. CCD-966SK cells were seeded at an initial density of 5 × 10^4^ cells/mL in a 24-well plate. After culturing for 24 h, the growth medium was replaced with normal medium (control), 5% dimethyl sulfoxide (DMSO), and the extract medium from CMC/PVA, CMC/PVA/CO-0.1%, CMC/PVA/CO-1%, CMC/PVA/CO-2.5%, CMC/PVA/CO-4%, and CMC/PVA/CO-5% groups. The extract media were prepared from the UV-sterilized composite membranes in the DMEM media supplemented with 10% FBS for 24 h. After 48 h of incubation, the morphology of CCD-966SK cells was observed under an optical microscope (Eclipse TS100; Nikon Corporation, Tokyo, Japan) and captured using a CMOS camera with SPOT Advance imaging software (SPOT Idea, Diagnostic Instruments, Sterling Heights, MI, USA). Cell viability was assessed by adding MTT (5 mg/mL) (Sigma-Aldrich, Burlington, MA, USA) to the cultured cells and incubating for 4 h. The supernatant was then removed, and 500 µL of DMSO was added to dissolve the formazan crystals. The absorbance (optical density, OD) of the purple formazan was quantified using a microplate reader (Model 2020, Anthos Labtec Instruments, Eugendorf, Wals, Austria) at wavelengths of 570/690 nm. Higher OD values indicated higher cell metabolic activity and hence better biocompatibility.

### 2.8. Antibacterial Activity Evaluation

The antibacterial activities of the CMC/PVA/CO composite films were assessed against Gram-positive bacteria (*S. aureus*, ATCC 25923) and Gram-negative bacteria (*E. coli*, ATCC 25922) using the disc agar diffusion method, as previously described [32]. The films were cut into 6 mm diameter round discs and disinfected under a UV lamp for 1 h. After disinfection, the films were gently placed onto the agar plates, which had been previously spread with bacterial broth cultures (100 μL). All of the agar plates were subsequently incubated at 37 °C for 24 h, and the diameters of the clear zones around the CMC/PVA/CO films were measured and recorded in millimeters.

### 2.9. Statistical Analysis

The experimental data were analyzed using one-way analysis of variance (ANOVA) (SPSS Inc., Chicago, IL, USA) and Tukey’s honest post hoc test. The difference was considered to be statistically significant when the *p* value < 0.05 (*) and the *p* value < 0.01 (**).

## 3. Results

### 3.1. Surface Characterization

**Figure 1:**
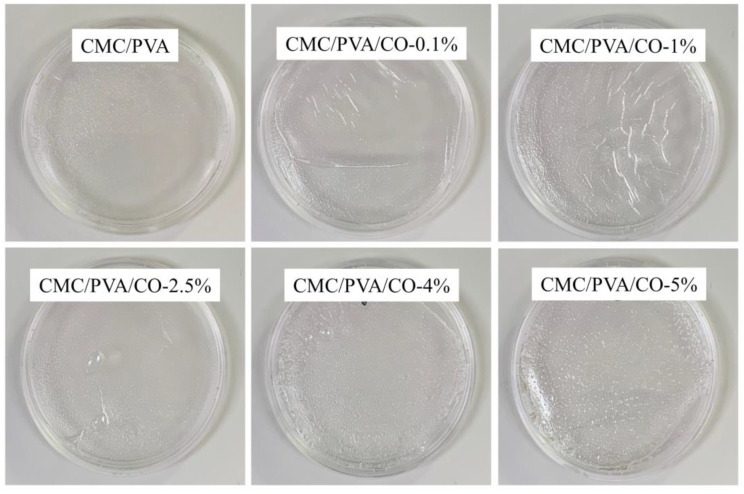
Visual appearance of the CMC/PVA/CO composite films.

Figure 1 shows the visual appearances of the CMC/PVA composite films containing different CO concentrations (0%, 0.1%, 1%, 2.5%, 4%, and 5%). All of the films show an integral and transparent appearance. With an increasing ratio of CO, rougher surfaces could be observed in the composite films, especially in the CMC/PVA/CO-4% and CMC/PVA/CO-5% groups.

**Figure 2:**
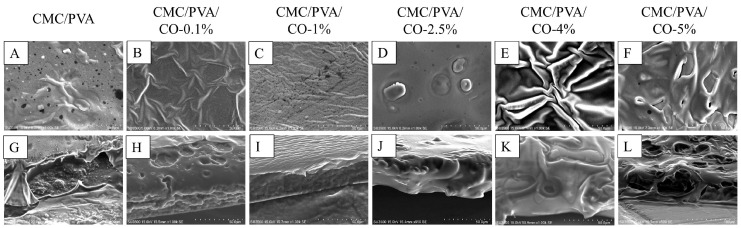
Scanning electron microscopy (SEM) micrographs of the CMC/PVA/CO composite films with different concentrations of CO (0.0%, 0.1%, 1%, 2.5%, 4%, and 5%): (**A**–**F**) surface morphology images, (**G**–**L**) cross-sectional images (magnification: 1000×).

A similar tendency is also observed in the SEM experiments (Figure 2). The surface morphology and structure of the CMC/PVA composite films is affected by the addition of CO (0%, 0.1%, 1%, 2.5%, 4%, and 5%). As shown in Figure 2, a smooth, continuous, and homogenous surface with fewer pores in the structure is observed in the CMC/PVA composite films with lower CO concentrations (0%, 0.1%, 1%, and 2.5%). This is because good compatibility and interactions exist between biopolymers and lower CO concentrations via hydrogen bonding between the –OH and –COOH groups, as confirmed by previous FTIR results [17,30]. However, the CMC/PVA composite films with higher CO concentrations (4% and 5%) exhibit surfaces with a certain unevenness and wrinkles. Moreover, the cross-sectional structure exhibits complex and irregular configurations, indicating that the film structure is significantly affected by the incorporation of higher CO concentrations and amounts of TWEEN-80 surfactant. Similar tendencies and results were also demonstrated in previous studies using higher levels of essential oils and surfactants [11,17,30].

### 3.2. XRD and FTIR Spectroscopy

**Figure 3:**
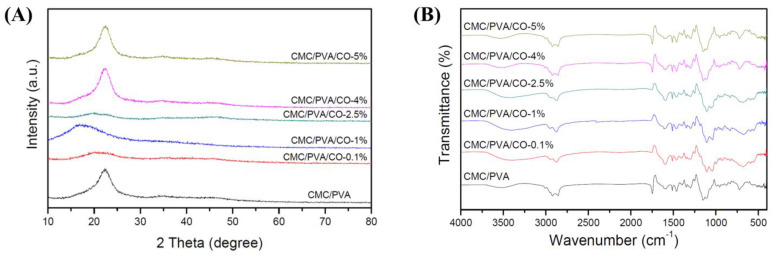
(**A**) XRD patterns and (**B**) FTIR spectra of the CMC/PVA/CO composite films with different concentrations of CO (0.0%, 0.1%, 1%, 2.5%, 4%, and 5%).

The X-ray diffraction (XRD) patterns show characteristic peaks, which identify the crystalline properties and phases of the prepared composite polymer films. Figure 3A shows the results of the XRD analysis. The diffraction pattern of pure CMC features a peak at 2θ = 22.20°, whereas that of pure PVA shows a significant peak at approximately 2θ = 19.5° [15,33]. The diffraction patterns of CMC/PVA composite films exhibit a peak at 2θ = 20°–25°, indicating that the strong interaction between CMC and PVA forms a composite [34]. These results are similar to those of previous studies [15,33]. Interestingly, with the addition of CO extract (0.1%, 1%, and 2.5%) to the CMC/PVA composite films, the crystallinity decreases and the amorphous phase increases because the diffraction peak becomes broad and weak. Moreover, this phenomenon may be due to the complete dissociation and successful miscibility between CMC and PVA after the incorporation of CO extract (0.1%, 1%, and 2.5%) [35]. However, the left shift of the broad peak in the CMC/PVA/CO-1% group may be associated with the changes in structure and the influence of pure PVA [15,33,34]. In addition, no new diffraction peaks are observed in the CMC/PVA/CO composite films, indicating that no chemical interaction occurs between CMC/PVA and CO.

The FTIR spectra of CMC/PVA composite films containing different CO concentrations (0%, 0.1%, 1%, 2.5%, 4%, and 5%) with their characteristic peaks are presented in Figure 3B. Wide bands located at 3000–3600 cm^−1^ are observed for all of the CMC/PVA/CO composite films. These bands are characteristic –OH stretching groups, which arise mainly from the strong hydrogen bonding between CMC and PVA. Sharp bands at 2900 cm^−1^ are also observed for all films, which are possibly related to the symmetric and asymmetric stretching of the CH_2_ groups of CMC and PVA [36,37]. Moreover, the peak at 1750 cm^−1^ is assigned to the carbonyl stretching of organic acids and phenolic compounds, which are the major components of CO [23,37]. Moreover, in a previous study, the major peaks of CO extract in FTIR spectra corresponded to hydroxyl groups (3488 cm^−1^), alkyl and C-H groups (2940 cm^−1^), methyl groups (1463 cm^−1^), ether groups (1230 cm^−1^), and terpenoid and flavone compounds (1049 cm^−1^), which also appeared in the CMC/PVA/CO composite films [28]. The –CH vibrational band is observed at 1330 cm^−1^, and the C-C stretching band is found at 835 cm^−1^. At 1650, 1473, and 1082 cm^−1^, symmetric and asymmetric stretching vibrations of C=O groups for CMC/PVA are observed [36,37]. Overall, similar major sharp peaks but varying peak amplitudes are observed in the FTIR spectra of the composite films, indicating that the incorporation of CO does not change the structure of the film matrix.

### 3.3. Thickness and Mechanical Properties

The thicknesses of the CMC/PVA, CMC/PVA/CO-0.1%, CMC/PVA/CO-1%, CMC/PVA/CO-2.5%, CMC/PVA/CO-4%, and CMC/PVA/CO-5% composite films are 58 ± 8, 58 ± 8, 50 ± 7, 88 ± 8, 128 ± 30, and 226 ± 32 μm, respectively. The thickness of the composite films increases with an increased CO concentration. Similar results were also reported in previous studies, where the thickness of composite films increased markedly with increasing concentrations of essential oils and extracts of polymer composite films [11,12,38].

**Figure 4:**
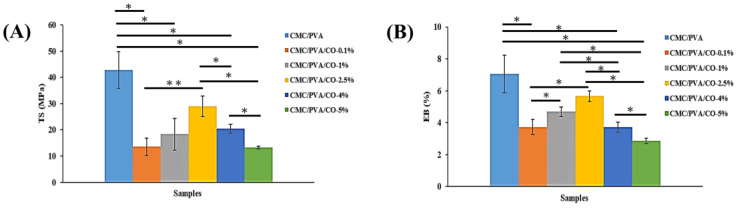
Mechanical properties of CMC/PVA/CO composite films with different CO concentrations (0.0%, 0.1%, 1%, 2.5%, 4%, and 5%): (**A**) tensile strength, (**B**) elongation at break rate. Results are expressed as mean ± standard deviation and statistical significance is represented as (*) *p* < 0.05 or (**) *p* < 0.01.

The TS and EB of the composite films are shown in Figure 4. As shown in Figure 4A, the TS of the pure CMC/PVA composite film is 42.8 ± 7.0 MPa. The TS decreases with an increasing CO concentration up to 5%, reaching a value of 13.2 ± 0.5 MPa. The TS of the CMC/PVA/CO-0.1%, CMC/PVA/CO-1%, CMC/PVA/CO-2.5%, and CMC/PVA/CO-4% composite films are 13.5 ± 3.3, 18.2 ± 5.9, 28.9 ± 3.8, and 20.4 ± 1.7 MPa, respectively. Among the CMC/PVA/CO composite films, CMC/PVA/CO-2.5% has the TS closest to that of skin tissue (28 MPa) [33]. This result is in agreement with previous studies, which indicated that the more essential oils or extracts were incorporated, the greater the decrease in the mechanical properties of the polymer composite films would be [11,12,38].

A similar tendency is also observed for CMC/PVA/CO composite films in the EB analysis (Figure 4B). The EB of the CMC/PVA is 7.0 ± 1.1 MPa. With the addition of CO, the EB of the CMC/PVA/CO composite film decreases. The EB of the CMC/PVA/CO-5% is 2.8 ± 0.1 MPa, while CMC/PVA/CO-2.5% maintains a higher EB value (5.6 ± 0.3 MPa). Multiple reasons, including the viscosity, thickness, intra-molecular bonding force, and microstructure, are related to this reduction in mechanical properties [11,12,38]. The observed changes are also in agreement with the SEM, XRD, and FTIR results. In general, the CMC/PVA composite films exhibit significantly decreased mechanical properties when CO is added. However, CMC/PVA/CO-2.5% can maintain approximately 65% and 80% of the TS and EB values, respectively, of CMC/PVA composite films. A possible reason for this increase may be that a CO concentration of 2.5% can maintain the interfacial interaction of the CMC/PVA composite without weakening the structure compared with the other concentrations.

### 3.4. Surface Wettability

**Figure 5:**
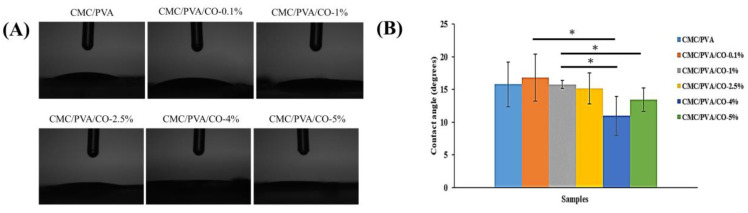
Contact angle analysis of the CMC/PVA/CO composite films with different CO concentrations (0.0%, 0.1%, 1%, 2.5%, 4%, and 5%): (**A**) digital images of the contact angles of water droplets on composite films, (**B**) average water contact angles of the composite films. Results are expressed as mean ± standard deviation and statistical significance is represented as (*) *p* < 0.05.

Surface wettability is an important material property for enhancing protein adsorption, the cellular response, and tissue repair. An improvement in surface wettability can promote cellular interactions between the prepared composites and living tissues [39,40]. Contact angle measurements of water droplets on the surface of the CMC/PVA/CO composite films were performed to evaluate the wettability. CMC and PVA are both highly hydrophilic polymers. Figure 5A shows digital images of the CMC/PVA/CO composite films. The contact angles of the CMC/PVA/CO composite films are 15.8° ± 3.4°, 16.8° ± 3.5°, 15.7° ± 0.6°, 15.1 ± 2.3°, 10.9° ± 2.9°, and 13.4° ± 1.8°, respectively (Figure 5B). In general, the contact angles of the CMC/PVA/CO composite films decrease with increasing CO concentration. The CMC/PVA/CO-4% exhibits the highest wettability of the composite films, whereas no significant differences are observed between CMC/PVA/CO-4% and CMC/PVA/CO-5%. Thus, the increased hydrophilicity of the CMC/PVA/CO composite films correlates with increasing CO concentrations. The surface roughness, structure, chemistry composition, and topographic design of the polymer composite films have great influence on wettability. In the present study, the improvement in surface wettability for CMC/PVA/CO-4% may be partially due to the heterogeneous surface geometries and structure [38,40]. Based on previous results, the increase in surface wettability of composite films would provide a desirable microenvironment for cell response, tissue engineering, and wound-healing applications [1,4,41].

### 3.5. Biocompatibility of the CMC/PVA/CO Composite Films

**Figure 6:**
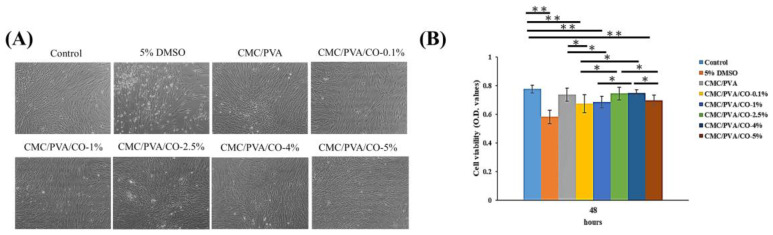
Biocompatibility evaluation of the CMC/PVA/CO composite films with different concentrations of CO (0.0%, 0.1%, 1%, 2.5%, 4%, and 5%) using the MTT assay. (**A**,**B**) Average cell viability values among the control, 5% DMSO, and composite films. Results are expressed as mean ± standard deviation and statistical significance is represented as (*) *p* < 0.05 or (**) *p* < 0.01.

Biocompatibility is essential for biomaterial applications [16]. Thus, the cytotoxicity of the prepared CMC/PVA/CO composite films was evaluated using the MTT assay. The cell morphology and cell viability of human dermal fibroblasts (CCD966SK) grown for 48 h under different culture conditions (control group, 5% DMSO, and CMC/PVA/CO composite film immersion medium) are presented in Figure 6. As shown in Figure 6A, human dermal fibroblasts cultured in the CMC/PVA/CO composite film immersion medium show good growth and healthy morphologies with minimal cell apoptosis compared to the 5% DMSO group. Based on the MTT assay data, all of the CMC/PVA/CO composite films exhibit comparable biocompatibility and non-toxicity. Thus, these composite films are suitable candidates for further tissue engineering and wound-healing applications. It has been reported that CMC/PVA films can absorb wound fluid through ion exchange, which enhances tissue formation, regeneration, and rapid epithelialization [5].

### 3.6. Antibacterial Properties of CMC/PVA/CO Films

**Figure 7:**
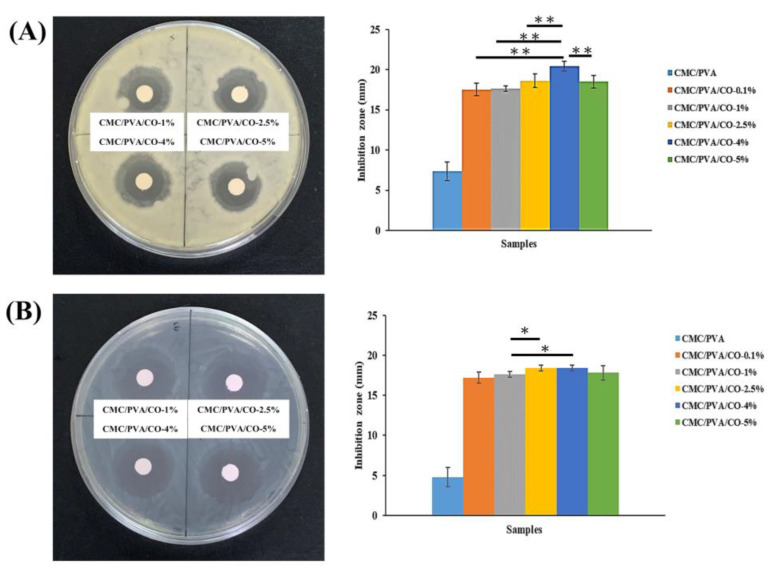
Inhibitory effects of CMC/PVA/CO composite films with different concentrations of CO (0.0%, 0.1%, 1%, 2.5%, 4%, and 5%) on the growth of (**A**) Gram-positive bacteria (Staphylococcus aureus) and (**B**) Gram-negative bacteria (Escherichia coli). Results are expressed as mean ± standard deviation and statistical significance is represented as (*) *p* < 0.05 or (**) *p* < 0.01.

*S. aureus* and *E. coli* are the major pathogenic bacterial strains in wound sites experiencing inflammation and chronic infection. Thus, *S. aureus* and *E. coli* are usually selected as representative bacteria to evaluate the antibacterial properties of composite films [34,37,42]. Figure 7 shows the antibacterial activity of the CMC/PVA/CO composite films against Gram-positive (*S. aureus*) and Gram-negative (*E. coli*) bacteria. In the absence of CO, the CMC/PVA composite film has lower antibacterial activity, while the CMC/PVA composite films containing various concentrations of CO exhibit antibacterial activity. With increasing CO concentration, the area of the inhibition zone increases. The diameters of the inhibition zones for CMC/PVA/CO-0.1%, CMC/PVA/CO-1%, CMC/PVA/CO-2.5%, CMC/PVA/CO-4%, and CMC/PVA/CO-5% composite films against *S. aureus* are 17.5 ± 0.7, 17.6 ± 0.3, 18.6 ± 0.8, 20.4 ± 0.6, and 18.4 ± 0.7 mm, respectively. Moreover, the diameters of the inhibition zones for CMC/PVA/CO-0.1%, CMC/PVA/CO-1%, CMC/PVA/CO-2.5%, CMC/PVA/CO-4%, and CMC/PVA/CO-5% composite films against *E. coli* are 17.2 ± 0.6, 17.6 ± 0.3, 18.4 ± 0.3, 18.4 ± 0.3, and 17.8 ± 0.9 mm, respectively. Overall, the ability of the CMC/PVA/CO composite film to inhibit *S. aureus* was better than its ability to inhibit *E. coli*. This is because the cell wall structure with a layer of peptidoglycan located between the outer membrane and the cytoplasmic membrane of Gram-negative bacteria is more complex than that of Gram-positive bacteria, which can protect bacteria from external stimulation [42]. These results are consistent with those of previous studies investigating CMC/PVA composite films incorporating nanoparticles, antibiotics, and essential oils [4,12,34,37,42]. The antibacterial effect of the pure CMC/PVA films is limited as shown in the present study and previous studies [36]. Thus, multiple strategies were developed to improve the antibacterial activity of the pure CMC/PVA films [4,12,33,37,42]. The antioxidant, antibacterial, and antifungal effects of CMC/PVA have been reported to be enhanced by the cooperation with 1.5% and 3.0% cinnamon essential oil [17,25]. This is the first study to show that the antibacterial effects of CMC/PVA composite films can be efficiently promoted by the addition of CO extract. According to previous studies, CO extract alone or in combination with polymer films or scaffolds can efficiently inhibit bacteria and enhance wound healing [27,28,43]. This is because polyphenols (especially flavonoids), which are the major constituents of CO, have excellent antimicrobial properties [21]. In addition, the major components of CO include carotenes, flavonoids, terpenoids, triterpenoids, polyphenols, phenolic acids, quinines, coumarins, carbohydrates, essential oils, minerals, and fatty acids [23,24,28]. The antimicrobial activity of CO has been attributed to flavonoids, triterpenoids, and essential oils. Possible mechanisms have been suggested for the antibacterial effects of CO, such as damaging the cytoplasmic membrane, denaturing proteins, disrupting the enzyme system, and reducing ATP synthesis [20,23,24,28,44]. Overall, the multifunctional characteristics of CMC/PVA/CO composite films are useful for wound-dressing applications because they contribute to wound healing at the stages of hemostasis, inflammation, protein adhesion, and cellular proliferation.

## 4. Conclusions

In the present study, CMC/PVA composite films containing various CO concentrations (0.1%, 1%, 2.5%, 4%, and 5%) were developed using a simple solution casting technique. The morphological, physical, mechanical, hydrophilic, biological, and antibacterial properties of the CMC/PVA composite films were affected by CO incorporation. The pure CMC/PVA composite film had smooth morphology and homogenous appearance, but CO incorporation resulted in a rough surface and irregular structure, especially regarding the CMC/PVA/CO-5% composite film. XRD and FTIR results indicate that CO incorporation does not change the structure of the film matrix. Results regarding the mechanical properties revealed that tensile strength and elongation rate upon the breaking of the composite films were influenced by CO incorporation. The addition of CO caused a decrease in mechanical strength, while the CMC/PVA/CO-2.5% composite films maintained approximately 65% of tensile strength and 80% of elongation rate at break. All composite films showed good hydrophilicity and that they were biocompatible towards the proliferation and growth of fibroblast cells. All concentrations of CO were effective against Staphylococcus aureus and Escherichia coli when compared to the pure CMC/PVA film. Finally, the improved physicochemical, biological, and antibacterial properties of the CMC/PVA/CO-2.5% composite films were found to be beneficial for further biomedical and wound-healing applications.

## Data Availability

The data presented in this study are available upon request from the corresponding author.
